# Relationship between Family and Myopia: Based on the Jiangsu School Student Myopia Study

**DOI:** 10.1155/2021/6754013

**Published:** 2021-07-14

**Authors:** Xiyan Zhang, Wenyi Yang, Jie Yang, Wei Du, Yao Xiang, Xin Wang, Chao Huang, Yan Wang, Fengyun Zhang

**Affiliations:** ^1^Department of Child and Adolescent Health Promotion, Jiangsu Provincial Center for Disease Control and Prevention, Nanjing, China; ^2^School of Public Health, Southeast University, Nanjing, China

## Abstract

**Purpose:**

This study aims to increase our understanding of the relationship between family and myopia in Chinese children.

**Methods:**

Students had a physical examination and were required to provide the necessary demographic information. Children and their guardians from different family types were required to fill in a questionnaire concerning myopia factors.

**Results:**

In this study, the prevalence of myopia in enrolled students aged 6–17 is 55.5%. The proportion of the nuclear family, extended family, single-parent family, and left-behind family is 40.6%, 43.7%, 11.1%, and 4.6%, respectively. Myopia rates from different family types by the order (nuclear family, extended family, single-parent family, and left-behind family) are 60.0%, 52.0%, 54.7%, and 50.9% taking on a decreasing trend, which shows an opposite trend comparing with elevated blood pressure, dental caries, and obesity. The interaction effect of the family type and region, physical examination, lifestyle (including diet habits, near work, outdoor activities, and sleep), and types of lamps and whether scolded by parents can have a significant impact on myopia. For primary school students (grade: 1–5), the prevalence of myopia in the nuclear family was a bit higher than that of myopia in the left-behind family, but for children in junior and senior high schools, both prevalences stayed similar.

**Conclusions:**

In this study, education pressure and time outdoors are still at play, and this kind of effect shows different phenomena in different families. Therefore, previous interventions would still work, and then the most critical challenge would be to ensure that left-behind children completed more schooling.

## 1. Introduction

Myopia is a significant public health concern and affects people worldwide and is estimated to almost 5 billion by 2050 [1, 2]. In China, a significant increase was also seen in the young generation, indicating the importance of the prediction of early-onset myopia among juveniles [3–5]. The prevalence of myopia in 2005, 2010, and 2014 Chinese National Students' Constitution and health survey indicated that myopia's peak prevalence has become earlier with age and kept a high level in children [6]. In 2018, the National Health Commission of the People's Republic of China had reported the prevalence of screening myopia for children from kindergarten (aged six years) to high school. The results were 14.5% for children aged six years, 36.0% for primary school students, 71.6% for middle school students, and 81.0% for high school students [7].

Family type, an important environmental factor, was reported to be associated with children's behavior [8]. Myopia is a complicated disease, and genetic as well as environmental factors contribute to its development [9]. Previously, we have demonstrated that environmental factors may play the leading role in forming myopia in Chinese children [10]. In a family, the parenting styles provided an environmental framework for children's psychosocial growth and were assumed to shape children's behavior [11]. Previously, we had reported that family type had an impact on elevated blood pressure [12]. However, studies on associations between family type and myopia were limited.

Therefore, this study examines if family type (nuclear family, extended family, single-parent family, and left-behind family) is associated with myopia for Chinese children. Also, it is aimed to increase our understanding of the relationship between family and myopia in Chinese children.

## 2. Methods

### 2.1. Study Design

This study is based on the project “surveillance for common disease and health risk factors among students” in Jiangsu Province, conducted during the 2018-2019 academic year.

### 2.2. Participants

We randomly selected schools in each of the 12 urban district/rural counties in Jiangsu Province and randomly sampled each school's students. In each of the selected schools, teachers, students, and doctors were included in this study. An autorefractor (Topcon RM-8900 or KR-800; Topcon Co., Tokyo, Japan) was applied with cycloplegia. The cycloplegic refraction is measured using tropicamide-phenylephrine eye drops every 5 min, three times. The refractive error is measured 30 min after the first drop of tropicamide by using autorefractor with five repeated measurements.

Meanwhile, students and their guardians were asked to fill in a questionnaire related to myopic information. The inclusion criteria for our subjects were as follows:

1. Nonmyopic children and lack of other serious eye diseases
2. Chinese Han nationality students
3. The ability of parents/guardians to provide informed consent

Detailed information can be seen in previous studies [10, 13].

### 2.3. Definitions

Myopia: it is defined as −0.50 diopters (D) in the worse eye, defined as the eye with the more excellent absolute value of refractive error (spherical equivalent) [14]. Anisometropia: significant anisometropia is often defined as a spherical equivalent, an interocular difference of ≥1.00 D. Astigmatism is reported as negative cylinder refraction ≥1.00 DC [15]. Premyopia: it is a refractive state of an eye of 0.75 D and >−0.50 D in children where a combination of baseline refraction, age, and other quantifiable risk factors provides a sufficient likelihood of the future development of myopia to merit preventative interventions [16].

Nuclear family: a family that consists of a father, mother, and children, when it is thought of as a unit in the society [17]. Extended family: a family group with a close relationship among the members including parents, children, and grandparents [18]. Single-parent family: the child living with a single parent that included divorced or widowed [19]. Left-behind family: children living with their grandparents only, and most of them were left-behind children [20].

### 2.4. Ethics Statement

The Institutional Review Board approved the Ethics Committee of Jiangsu Province CDC's study protocol, but there is no ID number for the approval. Reasons can be listed as follows: our study had no patients, did not involve the extraction of biological materials such as blood, pleural effusion, and cerebrospinal fluid (CSF) sampling, and had no experimental design. Next, this is our daily monitoring task to ensure students' health. The students and their parents were informed about the survey's aim, and teachers obtained participants' and their parents' oral and written consent. Detailed information can be found in the previous article [12].

### 2.5. Statistical Analysis

Descriptive statistics summarize the variable regarding the characteristics of Chinese students aged 6 to 17 years. Multiple regression analysis was performed, and a log odds ratio with 95% CI was computed to assess the relationship between common childhood disease and family type [21–23]. The data were analyzed using office software and SPSS V.20.0 software.

## 3. Results

### 3.1. Baseline Data

In this study, two thousand eight hundred forty-nine students are from the nuclear family accounting for 40.6%, and 3072 students come from the extended family accounting for 43.7%. There are 780 students (11.1%) in the single-parent family and 322 students (4.6%) in the left-behind family. The prevalence of elevated blood pressure, dental caries, and obesity from different family types varies significantly, e.g., the prevalence of elevated blood pressure, dental caries, and obesity from the nuclear family is lower than that of the left-behind family. The calculated odds ratio values of the relationship between family types and common student disease including elevated blood pressure, dental caries, and obesity are 0.37 (95% CI: 0.18–0.56), 0.05 (95% CI: −0.14–0.24), and 0.34 (95% CI: 0.17–0.51). Different regions show a different distribution of the rate, which may be related to the local economy, culture, education, and other factors (Table 1 and Supplement Figures 1 and 2).

### 3.2. Trends of Elevated Blood Pressure, Dental Caries, Obesity, and Myopia among Chinese Children and Adolescents by Different Family Types

In this study, the prevalence of enrolled students aged 6–17 years is 55.5%. The myopia rate of female students is 56.6%, and male students have a myopia rate of 54.6% (*P*=0.09). Children from the urban region (57.8%) have a higher prevalence of myopia than that of children (53.0%) from a rural region (*P* < 0.01). The prevalence of myopia among students from different families including nuclear family, extended family, single-parent family, and left-behind family is 60.0%, 52.0%, 54.7%, and 50.9%, respectively. The odds ratio values for the 95% confidence interval of the relationship between family types and myopia did not contain one suggesting that the family structure significantly affects myopia (Table 2 and Supplement Table 1).

### 3.3. Interaction Effect of the Family Type and Related Factors Such as Physical Examination and Lifestyle

The interaction effect of the family type and region (OR: 0.90; 95% CI: 0.82–0.99), father myopia (OR: 0.83; 95% CI: 0.74–0.94), mother myopia (OR: 0.77; 95% CI: 0.68–0.86), BMI (OR: 0.90; 95% CI: 0.88–0.91), lifestyle (including diet habits, near work, outdoor activities, and sleep), and types of lamps (OR: 1.16; 95% CI: 1.07–1.27) and whether scolded by parents (OR: 0.85; 95% CI: 0.81–0.89) can have a significant impact on myopia. This study investigated the related myopic behavior of children from different families, e.g., children from nuclear family and left-behind family exhibit different habits on turning on lights in class (*P* < 0.01), time spent on homework (*P* < 0.01), whether they have correct reading and writing gesture (*P* < 0.05), and screen time (pad/phone/TV) (*P* < 0.01). Nevertheless, two kinds of children had a similar outdoor activity time (*p*=0.37) (Table 3 and Supplement Figure 3).

### 3.4. Prevalence of Myopia between Nuclear Family and Left-Behind Family

**Figure 1:**
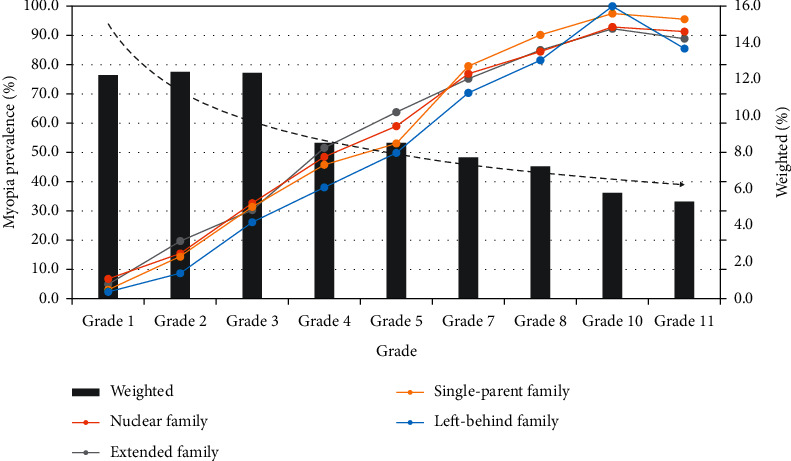
Prevalence of myopia of different grades in different family types.

In general, children in the nuclear family had a higher myopia prevalence than that of children in the left-behind family (*P* < 0.01). For primary school students (grade: 1–5), the prevalence of myopia in the nuclear family was a bit higher than that of myopia in the left-behind family, but for children in junior and senior high schools, both prevalences stayed similar. The weighted proportion of primary school students in this survey is high (Figure 1).

### 3.5. Prevalence of Premyopia, Astigmatism, and Anisometropia in Different Families

**Figure 2:**
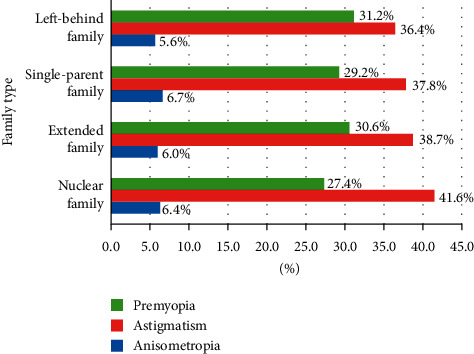
Prevalence of premyopia, astigmatism, and anisometropia in different family types.

The prevalence of premyopia and astigmatism varies among different family types (*P* < 0.05). In the meantime, the prevalence of anisometropia in different families was similar (*P*=0.824) (Figure 2).

## 4. Discussion

To the best of our knowledge, the present study is the first investigation to explore the relationship between family type and myopia among Chinese children. In this study, the prevalence of enrolled students aged 6–17 years is 55.5%. The prevalence of elevated blood pressure, dental caries, and obesity from different families, including nuclear family, extended family, single-parent family, and left-behind family, increased. However, the prevalence of myopia among students from different families including nuclear family, extended family, single-parent family, and left-behind family was 60.0%, 52.0%, 54.7%, and 50.9%, respectively, which took on a decreasing trend. For primary school students (grade: 1–5), the prevalence of myopia in the nuclear family was a bit higher than that of myopia in the left-behind family, but for children in junior and senior high schools, both prevalences stayed similar. The findings add to a small but essential set of studies implicating family type and myopia among Chinese children.

We found that children from different families shared different prevalences of myopia and other students' common diseases such as elevated blood pressure, dental caries, and obesity. It has been reported that family interventions in poverty, health, education, gender, and violence problems have proved effective and had a positive impact on health [24]. One study found that children in single-parent families and stepfamilies were more likely to experience hospitalization or an injury attributable to an accident than children living with biological parents [25, 26]. Children from a nuclear family and left-behind family exhibited different habits in many aspects. Parenting practices can influence some behaviors of a child, such as eating behaviors, violent behavior, and alcohol abuse [27–29]. The aforementioned findings suggested a link between family type and significant health outcomes in children, and a rising public health concern should be noted. However, this survey focuses on primary school students. In Supplement Figure 4, we added the results of myopia screening for people aged 12–17 in 2020. This part shows that the proportion of left-behind families is gradually decreasing in middle school, and the proportion of nuclear families is increasing. Meanwhile, the prevalence of screening myopia in a nuclear family is higher than that of a left-behind family. This phenomenon indicated that the most crucial challenge is to ensure that left-behind children completed more schooling and to pay more attention to vulnerable populations such as left-behind family children.

Besides, we found that the prevalence of premyopia and astigmatism varied among different families, and anisometropia developed similar among children from different family types. Premyopia is a refractive state of an eye of ≤0.75 D and >0.50 D in children where a combination of baseline refraction, age, and other quantifiable risk factors provides a sufficient likelihood of the future development of myopia to merit preventative interventions [16]. It is a useful concept that may promote further research among different family types. The family factor may be somehow contributing little to the formation of anisometropia, and studies have shown that the dominant eye may have a greater degree of myopia than the nondominant eye in subjects with anisometric myopia [30, 31].

Strengths and limitations of the study: the study's major strengths include the first study to explore the relationship between family type and myopia among Chinese students. Meanwhile, the current study presents associations between different family types and related myopic indexes, such as behaviors and premyopia. Limitations can be listed as follows: firstly, further study concerning the parental and children relationship should be conducted. Secondly, exploring precise intervention strategies on vulnerable populations would be our next job.

## 5. Conclusion

In this study, education pressure and time outdoors are still at play, and this kind of effect shows different phenomena in different families. Therefore, previous interventions would still work, and then the most critical challenge would be to ensure that left-behind children completed more schooling. We need to make a more specific analysis and precise intervention on vulnerable populations such as left-behind family children.

## Figures and Tables

**Table 1:** Demographic characteristics of the study of the Chinese children and adolescents.

	No.	%
Male	3829	53.5
Female	3332	46.5
Primary school	4408	61.6
Middle school	1590	22.2
High school	1163	16.2
Urban	3697	51.6
Rural	3464	48.4
Nuclear family	2849	40.6
Extended family	3072	43.7
Single-parent family	780	11.1
Left-behind family	322	4.6
Myopia	3976	55.5
Total	7161	100.0

**Table 2:** Trends of elevated blood pressure, dental caries, obesity, and myopia among Chinese children and adolescents by different family types.

Family type	Male/female	Elevated blood pressure (%)	Dental caries (%)	Obesity (%)	Myopia (%)
Nuclear family	1555/1294	13.8^#^	38.8^#^	18.3^#^	60.0^#^
Extended family	1646/1426	14.3^^^	46.6	19.5	52.0^^^
Single-parent family	388/392	12.7^+^	44.2	18.3^+^	54.7^+^
Left-behind family	169/153	18.7^#^+^	44.4^#^	23.6^#+^	50.9^#^+^
Total	3758/3265	14.1	43.1	19.1	55.5

^#^Nuclear family vs. left-behind family, *P* < 0.05. ^^^Extended family vs. left-behind family, *P* < 0.05. ^+^Single-parent family vs. left-behind family, *P* < 0.05.

**Table 3:** Effect of the family type interaction on myopia among Chinese children and adolescents.

Interaction	*β*	SE	Wald	*P*	OR (95% CI)
Urban-rural × family type	−0.10	0.05	3.92	0.04	0.90 (0.82–−0.99)
Father myopia × family type	−0.18	0.06	9.40	0.00	0.83 (0.74–−0.94)
Mother myopia × family type	−0.27	0.06	19.0	0.00	0.77 (0.68–−0.86)
Height × family type	0.00	0.00	0.32	0.57	1.00 (1.00–−1.00)
Weight × family type	0.04	0.00	205.83	0.00	1.04 (1.04–−1.05)
Systolic pressure × family type	−0.00	0.00	2.53	0.11	0.99 (0.99–−1.00)
Diastolic pressure × family type	0.01	0.00	7.96	0.01	1.01 (1.00–−1.01)
BMI × family type	−0.11	0.01	141.70	0.00	0.90 (0.88–−0.91)
Sweet food × family type	0.09	0.03	7.27	0.00	1.10 (1.03–−1.17)
Fresh fruits × family type	−0.08	0.02	14.75	0.00	0.93 (0.89–−0.96)
After-school study × family type	−.043	0.01	14.50	0.00	0.958 (0.94–−0.98)
Moderate/high-intensity exercise × family type	−0.03	0.01	25.13	0.00	0.98 (0.97–−099)
Outdoor activity at noon × family type	−0.18	0.09	3.88	0.04	0.83 (0.70–−0.99)
Doing eye-caring exercises × family type	−0.18	0.03	32.61	0.00	0.839 (0.79–−.89)
Digital screening × family type	0.06	0.03	5.49	0.02	1.06 (1.01–−1.11)
Sleep duration × family type	−0.06	0.02	8.55	0.00	0.94 (0.90–−0.98)
Sleep with light on × family type	−0.09	0.02	23.62	0.00	0.91 (0.88–−0.95)
LED × family type	0.15	0.04	12.10	0.00	1.16 (1.07–−1.27)
Scolded by parents × family type	−0.16	0.03	38.77	0.00	0.85(0.81–−0.89)

## Data Availability

All the relevant data are included within the manuscript, but original datasets cannot be shared because of involving students' personal privacy.

## Supplementary Materials

Supplementary Materials Supplement Figure 1: log odds ratio with 95% CI for the relationship between childhood common disease and family type. Supplement Figure 2: regional distribution of the study by different prevalences of the family type. Supplement Figure 3: relationship between myopia-related behavior and family type among Chinese children. Supplement Figure 4: association between screening myopia prevalence and family type among middle and high school students. Supplement Table 1: relationship between family type and myopia for children aged 6–17 years.
