# Supplementary material for: Relationship between Family and Myopia: Based on the Jiangsu School Student Myopia Study
Source: J Ophthalmol. 2021 Jul 14;2021:6754013. doi: 10.1155/2021/6754013 (PMC8298182; doi:10.1155/2021/6754013)
Supplement: Supplementary Materials — Supplement Figure 1: log odds ratio with 95% CI for the relationship between childhood common disease and family type. Supplement Figure 2: regional distribution of the study by different prevalences of the family type. Supplement Figure 3: relationship between myopia-related behavior and family type among Chinese children. Supplement Figure 4: association between screening myopia prevalence and family type among middle and high school students. Supplement Table 1: relationship between family type and myopia for children aged 6–17 years. [file 6754013.f1.pdf]

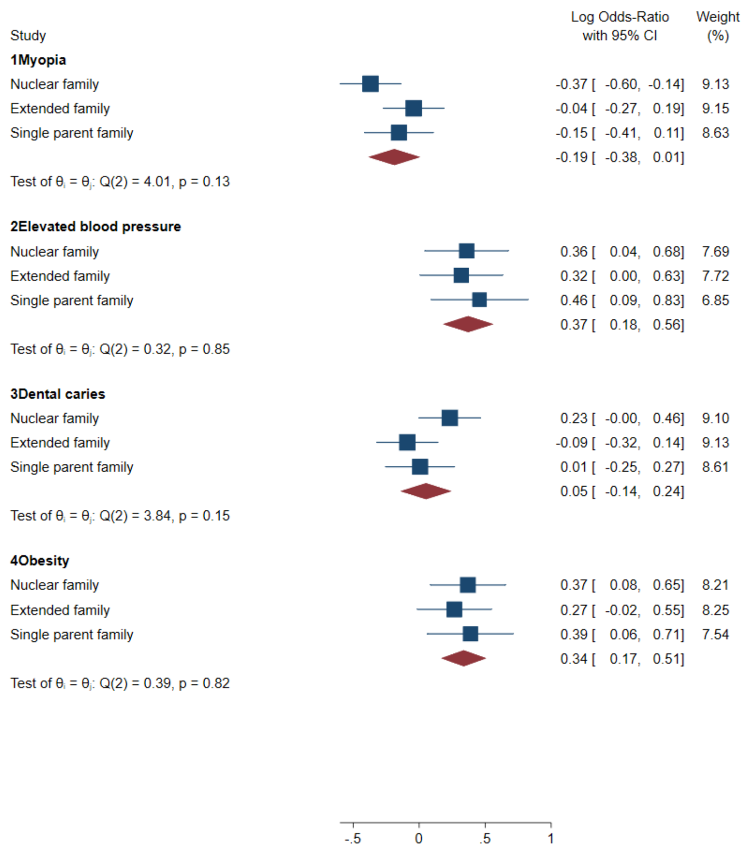

Supplement figure 1 Log odds-ratio with 95% CI for relationship between childhood common disease and family type

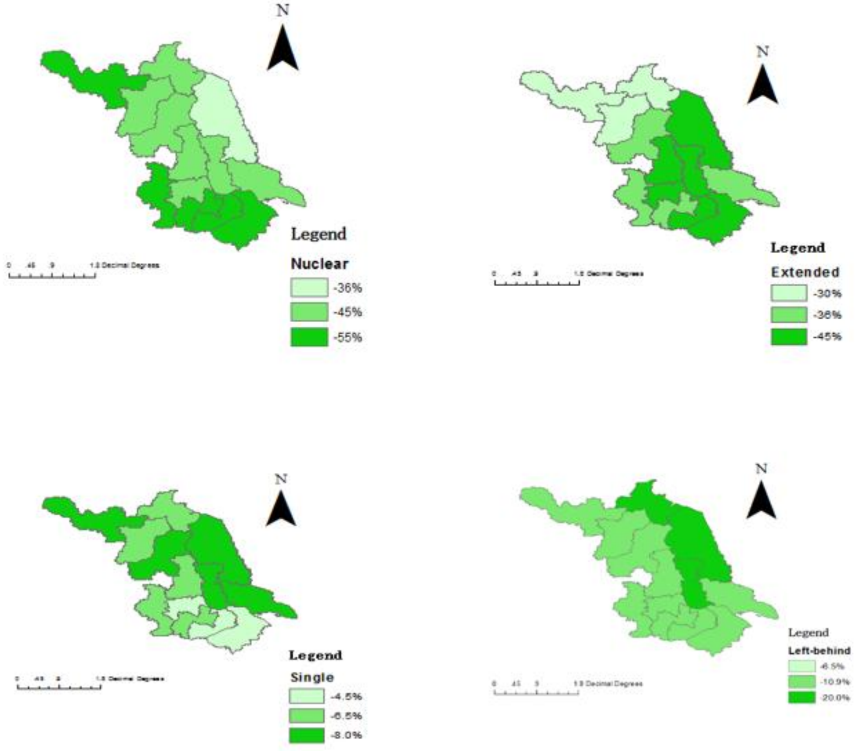

Supplement Figure2 Regional distribution of the study by different prevalence of the family type

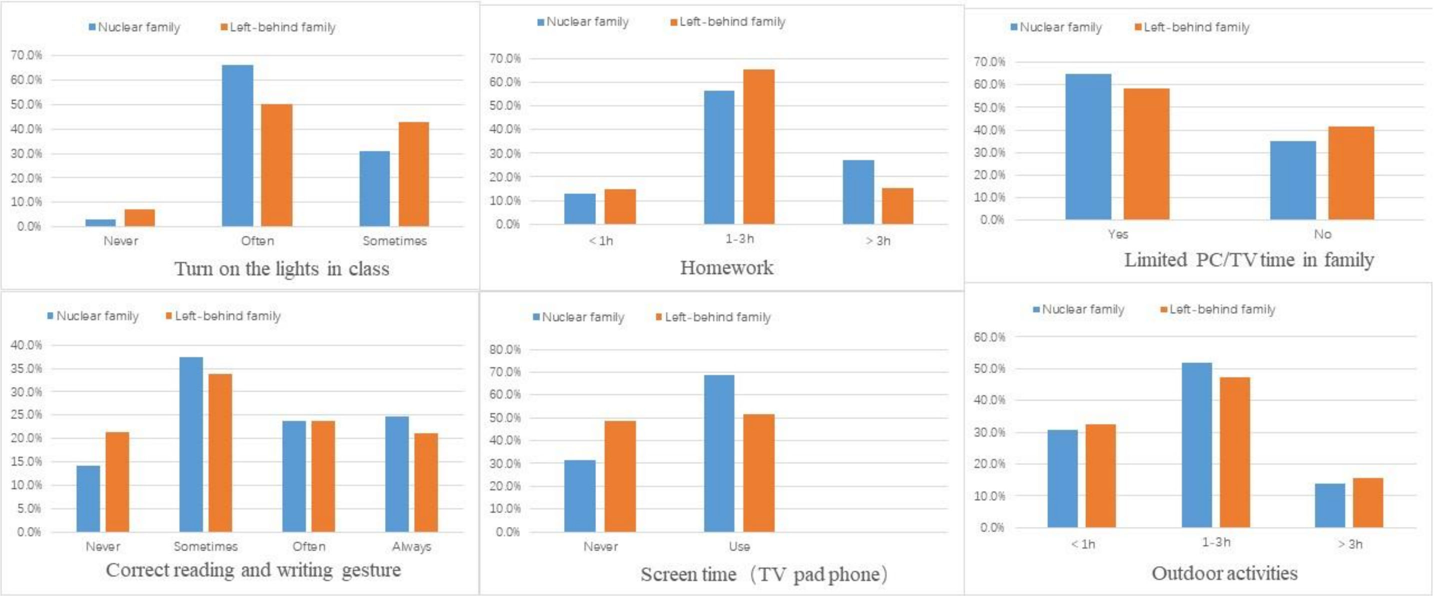

Supplement figure3 Relationship between myopic related behavior and family type among Chinese children

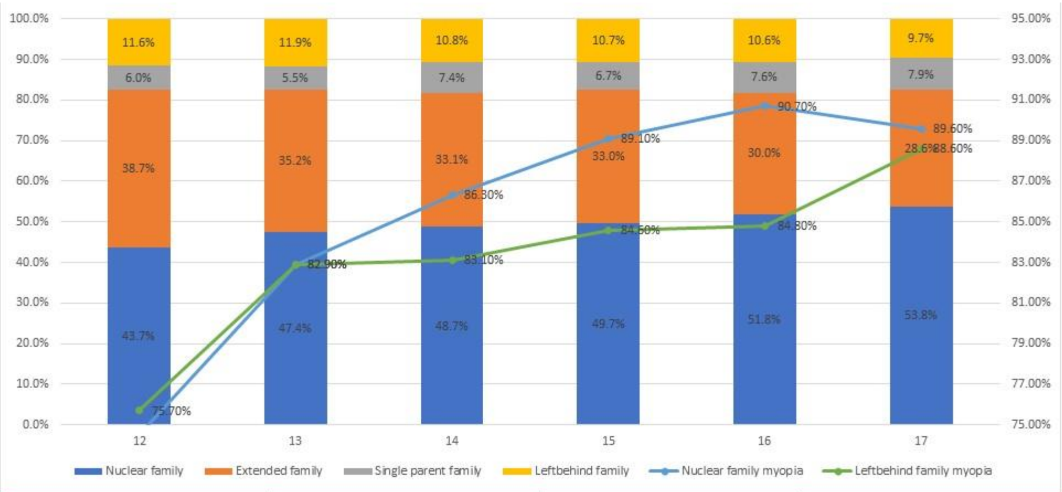

Age	Total	Nuclear family	Left-behind family
12-	4119	1798	477
13-	4077	1931	484
14-	4233	2063	456
15-	4303	2137	461
16-	4218	2186	448
17-	3707	1994	360

Supplement figure4 Association between screening myopia prevalence and family type among middle and high school students

Supplement table1 Relationship between Family type and myopia for children aged 6-17 years

Family type	Female-myopia (%)	Male-myopia (%)	Urban-myopia (%)	Rural-myopia (%)	Myopia (%)
Nuclear family	62.6	57.9	64.0	55.2	60.0
Extended family	52.1	51.8	51.7	52.2	52.0
Single parent family	53.1	56.4	60.2	48.8	54.7
Left-behind family	56.2	46.2	48.4	52.6	50.9
Total	56.6	54.6	57.8	53.0	55.5
